# School-Related Risk Factors of Internet Use Disorders

**DOI:** 10.3390/ijerph16244938

**Published:** 2019-12-06

**Authors:** Sophie Kindt, Carolin Szász-Janocha, Florian Rehbein, Katajun Lindenberg

**Affiliations:** 1 Institute for Psychology, University of Education Heidelberg, Keplerstraße 87, 69120 Heidelberg, Germany; kindt@ph-heidelberg.de; 2 Institute of Psychology, Heidelberg University, Hauptstraße 47-51, 69117 Heidelberg, Germany; carolin.szasz@uni-heidelberg.de; 3 Criminological Research Institute of Lower Saxony, Lützerodestraße 9, 30161 Hannover, Germany; florian.rehbein@kfn.de

**Keywords:** Internet use disorder, Internet gaming disorder, risk factors, school, social behavior, learning behavior, procrastination

## Abstract

A growing body of research focusing on the risk factors of Internet use disorder (IUD) underlines the effect of sociodemographic variables like age and gender or comorbid mental disorders on IUD symptoms. The relation between IUD symptoms and school-related variables has to date been insufficiently studied. The present study closes this gap by investigating the relation between school-relevant factors such as absenteeism, school grades, procrastination, school-related social behavior, and learning behavior and IUD symptoms in a high-risk sample. *n* = 418 students between 11 and 21 years of age (*M =* 15.10, *SD =* 1.97), screened for elevated risk of IUD, participated in the study. Sociodemographic data, school grades and absent days, Internet use variables (time spent online and gaming), as well as school-related psychological variables (procrastination, learning behavior, and social behavior) were assessed via self-report questionnaires. IUD symptoms were assessed with an adapted version of the German Video Game Dependency Scale (CSAS), which is based on the 9 criteria for Internet gaming disorder in the DSM-5. The instrument was adapted to include the assessment of non-gaming IUD symptoms. Taking the hierarchical structure of the data into account, a multilevel modeling approach was used to analyze the data. Procrastination, time spent online, and gaming were significant predictors of IUD symptoms at the individual level, whereas social behavior significantly predicted symptoms of IUD at the school level. In addition to previous findings on risk factors of IUD, this study indicates the importance of school-related factors in the development of IUD, especially psychological factors that play a role in the school setting. The early age of IUD onset and the high relevance of prevention of IUD at the school age underline the relevance of this finding.

## 1. Introduction

### 1.1. Definition and Diagnostic Criteria

Since the dissemination of the Internet in everyday life in the 1990s, researchers started to investigate the phenomenon of excessive or addictive Internet use. Young’s [1,2] early research first demonstrated that the intensive use of the Internet can become maladaptive. Some Internet users had difficulties to control their time spent online and reported higher problem severity than non-dependent users [1]. Today, there is a large body of research on the epidemiology, etiology, risk factors, consequences, and treatments of addictive video game and Internet use. Various terms have been used to describe the phenomenon, such as “video game addiction” [3], “compulsive Internet use” [4], “pathological Internet use” [5], “problematic Internet use” [6], “Internet addiction” [1], or “Internet use disorder” (IUD). In this paper, the latter term will be used to describe the addictive use of both video games and of other Internet applications (e.g., social media).

Many approaches have been developed over the past years in order to define IUD, resulting in a first uniform definition by the American Psychiatric Association in the 5th edition of the Diagnostic and Statistical Manual of Mental Disorders (DSM-5). The DSM-5 [7] includes “Internet gaming disorder” (IGD) in Section 3 (“conditions for further study”). Following the diagnostic criteria for substance-related addictions and pathological gambling, the DSM-5 proposes 9 criteria to define IGD:

1. Preoccupation with games
2. Withdrawal symptoms when not playing games
3. Tolerance, i.e., the need to spend increased amounts of time playing games
4. Unsuccessful attempts to stop or control gaming
5. Loss of interest in other recreational activities than gaming
6. Continuation of gaming despite psychosocial problems
7. Lying and deceit of others about the amount of gaming
8. Use of video games to escape from negative mood or real-life problems
9. Loss or risk of losing important relationships, career or educational opportunities as a result of gaming behavior.

The DSM-5 states that non-gaming IUD has not been sufficiently investigated to include it as a diagnosis [7]. However, recent studies indicate that non-gaming IUD entails similar levels of psychosocial strain as IGD [8]. The DSM-5 proposes to further study non-gaming IUD using similar guidelines as for IGD [7]. In the latest version of the International Classification of Diseases (ICD-11) [9,10], “gaming disorder” is included as a diagnosis under the newly included section of “behavioral addictions”.

In the ICD-11, “Gaming disorder is characterized by a pattern of persistent or recurrent gaming behavior (“digital gaming” or “video-gaming”), which may be online (i.e., over the Internet) or offline, manifested by:

(1) Impaired control over gaming (e.g., onset, frequency, intensity, duration, termination, context);
(2) Increasing priority given to gaming in the sense that gaming takes precedence over other life interests and daily activities;
(3) Continuation or escalation of gaming despite the occurrence of negative consequences.

The behavior pattern is of sufficient severity to result in significant impairment in personal, family, social, educational, occupational or other important areas of functioning. The patterns of gaming behavior may be continuous or episodic and recurrent. The gaming behavior and other features are normally evident over a period of at least 12 months in order for a diagnosis to be assigned, although the required duration may be shortened if all diagnostic requirements are met and symptoms are severe.” [10].

The ICD-11 offers the possibility to classify non-gaming IUD as “other specified disorders due to addictive behaviors.

### 1.2. Prevalence

Prevalence estimates vary greatly across studies due to lacking homogeneity of measurement instruments and non-representativeness of samples and range from 0.7% among adults in the United States [11] to 26.7% among adolescents in China [12]. Meta-analyses report a global prevalence estimate of 6.0% for IUD [13] and 4.6% for IGD among adolescents, respectively [14]. IUD seems to be more prevalent in younger age groups, with numbers up to 26.3% among adolescents and young adults in the United States [15], 6.3% in Chinese elementary and middle school students [16], and 4.4% to 6.9% among European and Israeli adolescents [17,18]. However, reported estimates have to be treated with caution, as there has to date been no gold standard for the definition of IUD. Moreover, prevalence studies often use screening questionnaires, which are by definition suited for screening and do not allow to establish a formal diagnosis [19]. Still, the numbers indicate that IUD is a significant problem, especially among adolescent populations.

### 1.3. Risk Factors

Besides young age, gender seems to be another important predictor of IUD. Several studies report a higher prevalence of IUD and especially IGD in males [20,21,22,23,24,25]. Other studies report equally distributed prevalence rates across gender regarding IUD [17,26,27]. When taking a more differentiated look, research indicates that males tend to display problematic video gaming more often (resulting in higher prevalence rates for IGD), while females are more prone to an excessive and maladaptive use of non-gaming Internet activities, especially social networking [8,27,28].

IUD is associated with high psychosocial strain [8,29] and comorbid psychiatric disorders are common. Specifically, IUD is often accompanied by attention-deficit/hyperactivity disorders (ADHD), depression, hostility and aggression, obsessive-compulsive disorders, anxiety, substance-related disorders, and personality disorders [30,31,32,33], regardless of whether the primary Internet activity is gaming, social networking, or other [27].

As children and adolescents spend large parts of their day at school, IUD also interferes with school-related factors, such as interaction with classmates, learning behavior, and academic performance. Since the adolescent age is the stage of life that shapes one‘s future significantly (e.g., academic career), these aspects are of particular importance. IUD is associated with poor academic achievement [21,22,31,34,35,36,37,38,39] and higher rates of absenteeism [21,22,38,40]. In addition, a low academic self-concept seems to be a risk factor for IGD [41]. Concerning the level of education, findings are mixed. Some studies reported higher rates of IUD among the higher educated [37,42], while others reported lower levels of education to be associated with IUD [22,43,44] or found no significant difference [21,25]. A possible explanation for low academic performance in students with IUD may be a lack of self-control [39,45,46,47]. Students who fail to control their own gaming and Internet behavior spend more time online and on video games. Although time spent online per se does not define addictive use, it is associated with gaming and non-gaming IUD [17,21,27,35,48]. Those students also tend to procrastinate on their schoolwork, resulting in lower grades. Procrastination has been found to be significantly associated with IUD in adolescents [49,50] and adults [51,52]. In line with findings of comorbid ADHD in individuals with IUD [30,53], increased impulsivity equally seems to be an associated factor [21,54]. Moreover, research on the delay of gratification has demonstrated a strong association between self-control and the ability to delay rewards in early childhood and academic and cognitive performance, but also social competencies and the ability to cope with stress in later life [55,56]. Duckworth and Seligman [57] found that self-rated self-discipline and the ability to delay a monetary reward in 8th-grade high-school students predicted academic performance at the end of the school year, above and beyond the effect of IQ. Furthermore, students with higher self-discipline had less absent days, spent more time doing their homework and started to do their homework earlier during the day. Thus, self-control seems to be crucial at school, but also in the development of psychosocial problems such as IUD.

Beyond academic achievement, psychological factors such as reduced well-being at school and poor social integration in the class are risk factors for the development of IGD [58]. Reduced social integration may be a result of reduced empathy and social competencies. Empathy has been found to be negatively associated with IUD in Chinese and German adolescents [59]. Poor social skills are associated with IGD [21,60,61]. On the other hand, social skills can be regarded as a protective factor [61]. Moreover, Mößle and Rebein [41] found peer problems and a low academic self-concept to be linked to problematic video game use.

To date, associations between school-related factors such as academic achievement, procrastination, absenteeism, social and learning behavior at school, and IUD remain insufficiently studied. The present analysis aims to close this gap by investigating the association between school-related factors and symptoms of IUD. Another purpose of this study is to explore the effects of sociodemographic variables, such as gender and age, as well as specific characteristics of the Internet use behavior (gaming vs. non-gaming) and the amount of time spent online. Specifically, we expect (1) a positive association between procrastination behavior and IUD symptom severity, (2) a negative association between social skills and IUD symptom severity, and (3) a positive association between deficient learning behavior and IUD symptom severity.

## 2. Materials and Methods

### 2.1. Sampling Procedure and Participant Characteristics

Data for the present study were collected in the context of the PROTECT study (ClinicalTrials.gov: NCT02907658), a longitudinal randomized controlled trial investigating the effectiveness of a school-based cognitive-behavioral group-intervention to prevent IUD. Ethical approval was obtained from the University of Education Heidelberg Research Ethics Committee on 3 September 2015 (Az.: 7741.35-13). Approval from the Regional Council was obtained on 19 October 2015 (Az.: 71c2-6499.25). Informed written consent was obtained from all participants as well as from their legal guardians. The study was conducted in line with the principles of the Declaration of Helsinki.

Data collection was conducted in schools during regular school hours between September 2015 and February 2018. The present study focuses on *n* = 418 participants in 34 schools in the area around Heidelberg, Germany, who completed the first assessment (baseline data). These students were between 11 and 21 years old (*M =* 15.10, *SD =* 1.97), *n* = 231 (55.3%) were female. We also included participants in their early adulthood in our study (18–21 years) because our sample included vocational schools that offer high school education for students who decided to take a higher-level education after or parallel to vocational training. Study participants attended different school types within the German school system, which can be classified into low, intermediate, and high educational levels. The largest part of the participants attended a school with a high educational level, aiming at matriculation in a university (“Gymnasium”; *n* = 271, 64.8%), *n* = 97 participants (23.2%) attended a school with an intermediate educational level (“Realschule”), and *n* = 50 participants (12.0%) attended a school with a low educational level (“Hauptschule/ Werkrealschule”). The average school grade in the studied sample (mean grade of German and Math) was *M =* 2.95 (*SD =* 0.74) within the German grading system (1 = “very good” to 6 = “insufficient”). On average, participants reported 1.47 sick days during the last 4 weeks (*SD =* 2.52). Regarding preferred Internet activities, *n* = 300 participants (71.7%) indicated that they used the Internet for “surfing” often or very often, *n* = 311 (74.4%) reported “chatting” as often or very often, and *n* = 144 (34.5%) used it for “gaming” often or very often.

The present sample was a high-risk sample defined by a cutoff of 20 points in the Compulsive Internet Use Scale (CIUS). The CIUS is composed of 14 items that can be rated on a 5-point Likert-scale ranging from 0 = “never” to 4 = “very often.” A total score between 0 and 56 can be obtained. The instrument has a good internal consistency (Cronbach’s α = 0.89–0.90 [62]. A total score of 24 and higher is recommended to identify cases with a sensitivity of 70% [63]. In our study, in order to achieve high sensitivity and simultaneously limiting the total number needed to treat, we used a more liberal cut-off criterion (CIUS ≥ 20) to select participants with elevated risk for IUD. The CIUS is a widely used diagnostic instrument that is suitable for the screening of IUD and to identify those individuals with increased risk.

**Figure 1:**
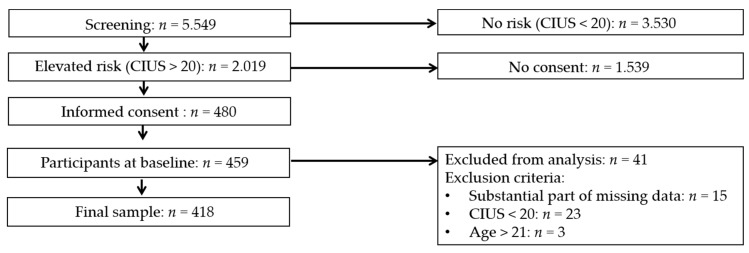
Flow chart of the study sample. CIUS: Compulsive Internet Use Scale.

Participants who were older than 21 years were excluded from the analysis, assuming that they did not show the typical characteristics of a high-school student population. Further criteria for the exclusion of cases from the analysis were substantial parts of missing data, a CIUS sum score below 20 and current treatment for IUD. Data collection was conducted by trained psychologists at school during school hours. The collection of the baseline data took approximately 45 minutes. Detailed sampling procedures are described elsewhere [64]. Figure 1 pictures a flow chart of the sample in this study.

### 2.2. Instruments

Within a longitudinal, randomized controlled study, participants completed a series of self-report questionnaires including data on sociodemography, overall psychopathology, IUD-specific symptoms, depression, anxiety, emotion regulation, procrastination, school-related social and learning behavior, self-efficacy, and psychological well-being. The relevant measures for the present study are described below. A detailed description of all measures can be consulted under the clinical trials registration page (ClinicalTrials.gov: NCT02907658) or in the PROTECT study protocol [64].

**IUD.** To assess IUD symptoms, we used an adapted version of the German Video Game Dependency Scale [22,65]. The original questionnaire covers the diagnostic criteria of IGD as defined in Section 3 of the DSM-5. Diagnostic criteria are covered by 2 items each, which can be rated on a 4-point Likert-scale from 0 to 3 (“not right,” “hardly right,” “rather right,” and “exactly right”). A diagnostic criterion is met if at least one corresponding item is rated with 3 (“exactly right”). A sum score can be computed and interpreted by grade- and age-specific norms. The instrument shows a high internal consistency reliability coefficient (Cronbach’s α = 0.94) and a high face validity due to its proximity to the diagnostic criteria of IGD [65]. To assess both IGD and non-gaming IUD symptoms, we adapted the CSAS items accordingly (e.g., “I feel that I can no longer control the time I spend on video games/*the Internet*,” for unsuccessful attempts to control). Because of previous research, indicating that non-gaming IUD showed similar characteristics as IGD [8], we considered an instrument assessing the DSM-5 criteria as an appropriate measure for IUD symptoms.

**Social and learning behavior**. To assess students’ self-concepts for school-related social and learning behavior, we used the German Student Assessment List for Social and Learning Behavior (SLB) [66]. The questionnaire includes 6 subscales for social behavior (24 items) and 4 subscales for learning behavior (16 items). Factor analyses proved a hierarchical structure with 10 first-order factors and 2 second order factors [67]. The scale for social behavior includes:

- Cooperation (behavior in group work, helping classmates),
- Self-perception (reflecting on individual misconduct in social interactions),
- Self-control (control of one’s behavior under negative emotions such as anger),
- Empathy (understanding behavior and feelings of other individuals, prosocial behavior such as consolation or encouraging others),
- Appropriate self-assertion (dispute resolution abilities, perception, and expressions of personal needs),
- Social contact (ability to establish relationships with peers).

The scale for learning behavior includes:

- Perseverance (ability to handle difficult tasks demanding high efforts),
- Concentration (ability to focus on a task under circumstances which impede cognitive performance),
- Independence (ability to solve tasks autonomously),
- Diligence (handling learning material such as books or pens with care).

Participants were asked to rate their behavior at school over the last 4 weeks on a 4-point Likert-scale (“never,” “rarely,” “sometimes,” “often”). Sum scores can be computed for each subscale and for social behavior and learning behavior in general. Separate norms exist for different age groups and for boys and girls. Retest reliabilities of the SLB-subscales ranged between *r =* 0.56 and *r =* 0.74, internal consistency ranges between α = 0.74 and α = 0.84 [66,67]. Convergent validity has been proven by positive correlations with school grades, teacher ratings and specific subscales of the Strengths and Difficulties Questionnaire [68], e.g., prosocial behavior [66,67]. For parsimonious reasons, we computed 2 sum scores (social behavior and learning behavior) by summing up the subscales, respectively.

**Procrastination.** To assess procrastination, we used the German general procrastination scale [69]. This 18-item instrument consists of 3 subscales: (1) procrastination (7 items, e.g., “I postpone important tasks until the last moment.”), (2) aversion to tasks (6 items, e.g., “I feel uncomfortable when I need to begin working on important tasks.”), and (3) preference for alternatives (5 items, e.g., “Before I start with an important task, I prefer dealing with other things first.”). Items can be rated on a 7-point Likert-scale (“never,” “hardly ever,” “rarely,” “sometimes,” “often,” “almost always,” “always”). The dimensional structure of the instrument was proven in exploratory and confirmatory factor analyses [69]. Mean scores can be computed for each subscale and interpreted by gender-specific norms. To cover a broad concept of procrastination including all 3 subscales, we computed an average score for procrastination by computing the mean value.

**School grades and absenteeism**. We asked participants to indicate their grades in Math and German on their last school report as well as the number of days they missed out on school within the last 4 weeks. School grades are indicated in the German grading system (1 = “very good” to 6 = “insufficient”). We computed the average of grades in Math and German to obtain a single indicator for academic performance.

**Time spent online, specific Internet activities, and the definition of “gamers”.** Participants were asked to indicate the number of hours they spend online on weekdays and on weekends. A total score for time spent online per day was computed [(2 × hours spent online on weekend days + 5 × hours spent online on weekdays)/7]. Furthermore, participants were asked to indicate how often they used the Internet for gaming, communication (social networking, messengers), or surfing (watching videos, searching for information) on a 5-point Likert-scale (“never,” “rarely,” “sometimes,” “often,” “very often”). Participants who indicated “often” or “very often” for gaming were classified as “gamers”.

### 2.3. Statistical Analyses

All statistical analyses were conducted with IBM SPSS Statistics for Windows, Version 25.0 (IBM Corp. Released, 2017). Hierarchical Linear Modeling (HLM) was chosen for the main analyses [70,71,72]. The benefit of this approach is that it takes hierarchical or clustered structures into account (e.g., students nested within different schools) and consequently allows observations to be dependent (students within school X may be more alike with each other compared to students of school Y because of any contextual factor). Furthermore, HLM enables interpreting effects at different levels, e.g., individual effects on level 1 versus contextual effects on level 2. We used a 2-level-approach where individuals (level 1) were nested within schools (level 2). Model testing was performed in successive phases (empty model, random-intercept-random-slope models, random-intercept-fixed-slope-models) in accordance with the recommendations by Raudenbush and Bryk [73].

Given that the psychological predictor variables (procrastination, social behavior, learning behavior) as well as demographic and Internet use variables (age and the amount of time spent online) have no meaningful zero points and because we aimed to assess the effects of person-level predictors, we conducted the analyses using centering within clusters for level 1 predictors [74]. Level 2 predictors (school means) were grand mean centered before including them in the analysis.

## 3. Results

### 3.1. Descriptive Statistics

Table 1 pictures descriptive statistics for the studied variables. Internal consistency coefficients were computed for all scales and subscales. Internal consistency coefficients (Cronbach’s α) ranged from acceptable to excellent in our sample. In further analyses, total scores for social behavior and learning behavior and an average score for the procrastination scale were used as independent variables.

### 3.2. Correlation Analysis

To determine which predictor variables should be included into the hierarchical linear model, we computed intercorrelations between the group-centered independent variables (gaming vs. non-gaming, age, gender, school grades, sick days, social behavior, learning behavior, and procrastination) and the uncentered dependent variable (CSAS Sum Score). Table 2 displays the intercorrelations of the variables.

The results of the correlation analysis showed significant correlations between CSAS scores and gender (*r =* −0.15, *p =* 0.002), gaming (*r = 0*.27, *p <* 0.001), time spent online (*r = 0*.14, *p =* 0.004), procrastination (*r =* 0.26, *p <* 0.001), social behavior (*r =* −0.20, *p <* 0.001), and learning behavior (*r =* 0.23, *p <* 0.001). Furthermore, gender was significantly associated with gaming (*r =* −0.57, *p <* 0.001). Additionally, significant but small correlation could be observed between time spent online and procrastination (*r =* 0.11, *p =* 0.033), social behavior (*r =* 0.12, *p =* 0.016), and learning behavior (*r =* 0.14, *p =* 0.005), as well as between school grades and procrastination (*r =* 0.15, *p =* 0.003), social behavior (*r =* −0.20, *p <* 0.001), and learning behavior (*r =* −0.28, *p <* 0.001). Procrastination was furthermore significantly correlated to social behavior (*r =* −0.27, *p <* 0.001), as well as learning behavior (*r =* −0.48, *p <* −0.001), and social and learning behavior were intercorrelated (*r =* 0.53, *p <* 0.001). These findings, however, need to be interpreted with caution, as *p*-values are not Bonferroni-corrected and the hierarchical structure of data is not taken into account. We computed an OLS regression analysis to test the data for multicollinearity. As no correlation was *r* ≥ 0.7 and all values for tolerance were < 0.1, we concluded that multicollinearity was not an issue.

Only those predictors were tested in the HLM that were significantly correlated to the outcome: gender (*r =* −0.15, *p =* 0.002), gaming (*r =* 0.27, *p <* 0.001), time spent online (*r =* 0.14, *p =* 0.004), procrastination (*r =* 0.26, *p <* 0.001), social behavior (*r =* −0.20, *p <* 0.001), and learning behavior (*r =* 0.23, *p <* 0.001). All other variables (school grades, absent days, and age) were not significantly correlated to the outcome variable and were therefore not included in the HLM.

### 3.3. Multilevel Analysis

#### 3.3.1. Empty Model (Model 0)

To test the multilevel structure of our data, we first compared an empty model without a hierarchical data structure (0a) to one taking the hierarchical structure of the data into account (0b). The model fit was significantly better for the model including a hierarchical data structure (–2 Log Likelihood for 0a: *–2LL* = 2850.461; –2 Log Likelihood for 0b.: *–2LL* = 2843.441, *χ*^2^(1) = 7.02, *p <* 0.01). The Intraclass Correlation Coefficient was *ICC* = 0.062. Thus, 6.2% of the outcome variance were attributable to level 2 variation, i.e., differences between schools.

#### 3.3.2. Model Specification (Model 1–4)

The model was specified step by step as recommended by Raudenbush and Bryk [73], starting by including level 1 predictors with random components (random intercepts and random slopes) and then fixing those effects that did not substantially explain variance in the model. First, all predictors that were significantly correlated to the outcome, were tested separately with allowing slopes and intercepts to be random and then compared to a model in which slopes were fixed and intercepts were random.

The random-intercept-random-slope model for gaming did not converge, therefore slopes for gaming were fixed. For procrastination, the slope did not vary significantly across participants, i.e., model fit between the model with random slopes and the model with fixed slopes was minimal and non-significant (Var(u1j) = 0.06; *Δ-2LL* = *χ*^2^(1) = 0.01; *p >* 0.05), hence slopes for procrastination were fixed as well. The same was true for learning behavior (Var(u1j) = 0.0005; *Δ-2LL* = *χ^2^*(1) = 0.002; *p >* 0.05), social behavior (Var(u1j) = 0.006; *Δ-2LL* = *χ*^2^(1) = 0.45; *p >* 0.05), time spent online (Var(u1j) = 0.16; *Δ-2LL χ*^2^(1) = 1.40; *p >* 0.05), and gender (Var(u1j) = 0.81; *Δ-2LL χ*^2^(1) = 0.16; *p >* 0.05), hence slopes for learning behavior, social behavior, procrastination, time spent online, and gender were fixed as well. Gaming [*F*(1,315.58) = 28.45, *p <* 0.001], procrastination [*F*(1,27.06) = 32.12, *p <* 0.001], learning behavior [*F*(1,384,02) = 23.75, *p <* 0.001], social behavior [*F*(1,384.14) = 19.20, *p <* 0.001], gender [*F*(1,385.62) = 7.81, *p =* 0.005], and time spent online [*F*(1,371.22) = 8.79, *p =* 0.003] significantly predicted IUD symptoms in the separate models.

As none of the variables produced significant variability in slopes, a random-intercept-fixed-slope model with all these potential predictor variables was computed (Model 1). As we did not have any a priori assumptions about interaction effects and in order to keep the model as parsimonious as possible, only the main effects were tested. Model fit (indicated by –2 Log-Likelihood and Akaike Information Criterion) was compared for each model to its previous model and –2 Log-Likelihood was tested for significance using *χ^2^* tests. Pseudo R^2^ statistics were computed for level 1 and level 2 using the simplified formula by Snijders and Bosker [75]. The results of the HLM (Model 0 – Model 4) are presented in Table 3.

Model 1 showed that gaming [*F*(1,399.37) = 12.71, *p <* 0.001], procrastination [*F*(1,369.80) = 17.52, *p <* 0.001], and time spent online [*F*(1,368.16) = 4.40, *p =* 0.04] on the individual level significantly predicted the outcome when being investigated together. On average, Model 1 explained R^2^ = 14.98% of the outcome variance on level 1 and R^2^ = 12.27% on level 2. Model fit was significantly improved by adding those predictors as compared to the empty model (Model 0) (*χ^2^*(6) = 178.33 **; *p <* 0.01).

Next, we computed an intermediate reduced model including only those level-1 variables that were significant predictors of the outcome, namely gaming [*F*(1,403.83) = 25.79 *p <* 0.001], procrastination [*F*(1,368.61) = 29.23, *p <* 0.001], and time spent online [*F*(1,367.65) = 4.77 *p =* 0.030] on the individual level (Model 2). Model 2 explained R^2^ = 13.96% of the outcome variance on level 1 and R^2^ = 11.85% on level 2. Model fit decreased significantly in comparison to Model 1 (*χ*^2^(3) = −19.102, *p <* 0.01). This decrease was expected as the model contained less parameters.

Then, level 2 variables (group means of the continuous level-1-variables procrastination, learning behavior, social behavior, and time spent online, centered around the grand mean) were included in the model (Model 3). In addition, the academic level was included as a control variable. Model 3 again showed that gaming [*F*(1,351.28) = 25.40, *p <* 0.001], procrastination [*F*(1,368.77) = 28.77, *p<* 0.001], and time spent online [*F*(1,366.68) = 4.74, *p =* 0.030] on the individual level significantly predicted the outcome. At the school level, only the mean social behavior of the school was significantly related to the outcome [*F*(1,34.27) = 4.31, *p=* 0.046]. Thus, the average social behavior within a school significantly predicted IUD symptoms. Model 3 explained R^2^ = 18.36% of the variance at the individual level and R^2^ = 24.66% at the school level. Although the difference in model fit between Model 2 and Model 3 was not statistically significant (*χ*^2^(5) = 10.64, *p >* 0.05), Model 3 included another significant predictor at the school level.

Finally, a reduced model with only significant level-1 and level-2 predictors was computed (Model 4). In this final model, gaming [*F*(1,362.69) = 24.77, *p <* 0.001], procrastination [F(1,361.01) = 28.54, *p <* 0.001], and time spent online [F(1,358.62) = 4.75, *p =* 0.30] significantly predicted the outcome on the individual level and mean social behavior of the school predicted the outcome on the school level [*F*(1,21.99) = 9.64, *p =* 0.005]. Model 3 and Model 4 did not significantly differ in their model fit (*χ^2^*(3) = −4.00, *p >* 0.05). Model 4 explained R^2^ = 17.45% of the variance at the individual level and R^2^ = 23.00% at the school level. Model 4 was regarded as the final model as it included all relevant predictor variables and explained more variance than Model 2.

**Figure 2:**
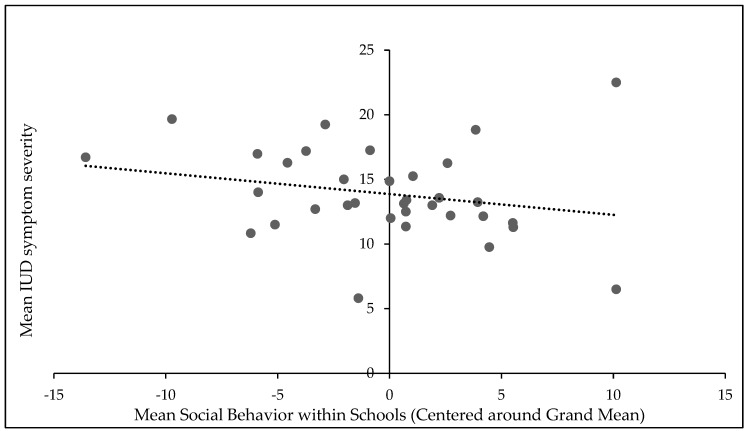
Mean social behavior within schools predicts mean IUD symptom severity. Scores for mean social behavior are centered around the grand mean.

Figure 2 pictures the relationship between mean social behavior within schools and mean CSAS scores, indicating that mean social behavior on the school level predicts average IUD symptom severity.

## 4. Discussion

This study gives insight into the relevance of school-related risk factors of IUD, carefully considering factors related to the students’ individual behavior and factors related to the school environment. Some expected risk factors found in other studies could not be confirmed here, as for example school absence and school grades were not associated with our main outcome and thus could not be investigated any further.

Taking individual school-related behaviors into account, procrastination stands out as a highly relevant risk factor for IUD symptoms in adolescence, confirming the results of other studies [49,50]. Procrastination is closely related to some clinical core symptoms of IUD. For example, problematic users tend to experience preoccupation and withdrawal symptoms in times they are engaged in online activities and are more prone to use the Internet in order to escape from negative moods or real-life problems. Thus, it could be assumed, that the tendency to postpone learning activities is closely related to the rewarding features of online and gaming activities. Unlike working on school-related tasks, which could be exhausting and might even evoke unpleasant feelings such as the sense of inferiority or fear to fail, students usually get instantly rewarded using social networks, games or other preferred online activities. As students usually have constant access to online activities through smartphones and computers, these rewarding experiences are permanently available and easy to achieve. Thus, online activities could not only distract students from school-related obligations in the short term but might also obstruct the completion of long-term learning goals due to the time-consuming character of many online activities. The more students tend to postpone activities for school in favor of their online behavior, the harder it might become to start working on them later, as obligations might accumulate, and negative feelings related to them might intensify.

Besides procrastination, we also expected that students with deficient learning and social behavior, in general, would exhibit a higher risk for IUD. As expected, social and learning behavior showed significant negative correlations with IUD symptoms, although a higher positive correlation could be observed between procrastination and IUD symptoms. In the HLM, however, only procrastination remained as significant predictor. For learning behavior and procrastination, it could be noted, that both variables share common variance and are at least moderately correlated (*r* = −0.48). Thus, procrastination can be regarded as one central aspect of learning behavior and might, in fact, be the most relevant one, when it comes to the risks for IUD. The scale used in this study for the measurement of learning behavior rather focusses on a self-assessment of certain learning abilities such as perseverance, concentration, independence, and diligence [67]. Thus, it could at least be speculated, that not learning abilities per se but bad learning habits such as procrastination could elevate the risk for IUD. For social behavior, however, it remains uncertain why deficits in social behavior on the individual level did not predict IUD symptoms in our study. We found a moderate correlation between social behavior and the learning behavior scale (*r* = 0.53), indicating that they are related. Thus, deficient learning habits might be associated with poor social behavior in class. The relationship between social behavior and IUD symptoms, in general, must be regarded as understudied. However, prior work has found poor social skills to be at least of relevance for IGD [21,60,61]. Thus, this might not be true for certain subtypes of dysfunctional online behavior, especially problematic social networking, which in fact is a social activity and might even train some social skills. Unfortunately, we cannot confirm nor disconfirm this assumption, as we only assessed IUD symptoms in general in our study.

Besides procrastination on the individual level, elevated time spent online increases the risk whereas being a non-gamer decreases the risk of IUD symptoms. IGD is currently considered the most relevant and best-studied subtype of IUD and will be recognized as an official disorder in the ICD-11. Gaming activities, especially the use of game genres predominantly played online, such as massively multiplayer online role-playing games, online-shooters, and online-strategy-games are associated with IGD [21,43,76]. In our correlation analysis, we found gender to be moderately correlated with gaming (*r* = 0.57). This is in line with previous studies showing a male predominance in gaming [8,24,28]. Our study shows, that even if gender and time spent online are controlled for, being a non-gamer significantly reduces the risk of IUD symptoms, again substantiating the relevance of gaming behavior for symptom severity. This result contradicts the findings of Strittmatter et al. [8] that pointed to similar symptom severity in gamers and non-gamers and is in line with the DSM-5 and ICD-11 approaches that only include gaming-related IUD as a diagnosis.

At the school level, social behavior remains as the only relevant risk factor predicting IUD symptoms. As we also controlled for the education level, this result could not be attributed to lower social behavior in lower education schools. Other environmental factors, such as general procrastination, learning behavior at school, or fellow students’ engagement in online activities are of no importance. Our finding is in line with other studies, showing that social problems and conflicts, such as peer problems and poor social integration in the class, could be the most relevant environmental school factors for IGD [41,58]. Social behavior at school might also be connected to other school-related risk factors associated with IGD, such as school-related well-being, school-related anxieties, and the academic self-concept of a student. In the extreme, problematic social behavior in the school could also facilitate bullying behavior among students and could lead to avoidance of school in general and other forms of school-associated problems regarding learning and academic achievement. This result suggests that prevention measures regarding health topics and addiction in the school context should include measures to promote prosocial behavior at school.

To sum up, the results of our study emphasize that school-related risk factors, such as the social school environment and deficient learning habits, i.e., procrastination, could be considered as not only of relevance for academic success but also for IUD symptoms in adolescence. Combined with elevated time spent online and frequent gaming activities, a risk profile is characterized, in which online and especially gaming activities are used not only for easy and instant reward-experiences but also to compensate and forget about social conflicts at school and demanding school obligations.

This study has some strengths and some weaknesses. Firstly, on the strong side, the sample was comprised of preselected high-risk students. Thus, it was possible, to use a smaller sample size to analyze the possible relevance of risk factors for IUD symptoms. Secondly, based on the literature, we used selected and validated formal paper-pencil-measures reflecting different school-related risk factors for IUD symptoms. Thus, it was possible to include not only individual school-related risk factors but also risk factors concerning the social school environment. Some limitations have to be considered: Firstly, data is cross-sectional as we used only the basement assessment of the longitudinal study. It was not possible to include data from later assessments, as it was influenced by the following intervention (CLINICALTRIALS.gov: NCT02907658). That means that no information on causality could be derived from the data analyzed in this study. As some of the risk factors included could also be considered as possible consequences of IUD, a longitudinal perspective would have strengthened our analyses. Secondly, because of our sampling strategy using the CIUS to pre-select risky online users, problematic gaming was not assessed separately from other subtypes of IUD. It would certainly have been of interest to differentiate between subtypes of IUD. The preselection of our sample can be regarded as a third limitation more generally, as it only allows inferences to a high-risk population. Fourthly, as we did not use in-depth diagnostic interviews but questionnaire assessments only, we can only draw inferences on risk factors for IUD symptoms, not diagnoses, from our results.

## 5. Conclusions

- To investigate the relevance of school-related risk factors of IUD symptoms, factors related to the students’ behavior and factors of the school environment should be differentiated.
- In the spectrum of variables describing the learning and social behavior of the individual, procrastination stands out as the most relevant risk factor of IUD symptoms.
- In the spectrum of variables describing learning and social behavior at the school level, the social behavior of students stands out as the most relevant risk factor of IUD symptoms.
- Universal prevention measures in the school context should also facilitate social competence and positive social interactions among students. They could also promote competences in dealing with stress and achieving long-term goals. Selective prevention measures might target specific groups of students exhibiting deficits in these respective areas.
- Future studies should more thoroughly address the longitudinal relationship between procrastination and certain subtypes of IUD.

## Figures and Tables

**Table 1:** Descriptive statistics and internal consistency coefficients.

Scale	Subscale	*M*	*SD*	Cronbach’s α
CSAS Sum Score		13.55	7.33	0.816
Social behavior	Cooperation	9.46	2.41	0.812
	Self-perception	9.24	2.29	0.762
	Self-control	8.34	2.80	0.837
	Empathy	9.53	2.58	0.846
	Appropriate self-assertion	9.31	2.29	0.767
	Social contact	8.80	2.71	0.804
	Total score	54.87	10.26	0.765
Learning behavior	Perseverance	7.45	2.65	0.819
	Concentration	8.23	2.61	0.865
	Independence	9.64	2.24	0.778
	Diligence	8.80	2.99	0.836
	Total score	34.20	8.12	0.772
Procrastination	Procrastination	4.13	1.30	0.897
	Task aversion	3.46	1.20	0.840
	Preference for alternatives	4.03	1.20	0.814
	Average score	3.87	1.11	0.938

Note. CSAS: German Video Game Dependency Scale; SLB: German Student Assessment List for Social and Learning Behavior.

**Table 2:** Intercorrelations of independent and dependent variables.

Variable	1	2	3	4	5	6	7	8	9	10
1. CSAS		−0.15 **	0.05	0.27 **	0.14 **	0.03	0.04	0.26 **	−0.20 **	−0.23 **
2. Gender			−0.6	−0.57 **	0.12 *	0.07	−0.08	0.08	0.13 *	0.04
3. Age				0.06	0.24 **	0.05	0.15 **	0.13 *	0.02	0.04
4. Gamer					0.12	−0.01	0.08	0.03	−0.11 *	−0.08
5. Time spent online						0.10 *	0.10	0.11 *	−0.12 *	−0.14 **
6. Absence							0.00	0.06	−0.09	−0.05
7. School grade								0.15 **	−0.20 **	−0.28 **
8. Procrastination									−0.27 **	−0.48 **
9. Social behavior										0.53 **
10. Learning behavior										

Note. * *p =* 0.05, ** *p =* 0.01. School grades are indicated in the German grading system (1 = “very good” to 6 = “insufficient”) and represent the average of the grades in Math and German. Age, time spent online, absent days, school grades, procrastination scores, and scores of social behavior and learning behavior were group-centered before being entered in the analysis. CSAS: German Video Game Dependency Scale. Gender: male = 0; female = 1.

**Table 3:** Results of the multilevel analysis.

	IUD Symptoms (CSAS Sum Score)				
Parameter	Model 0	Model 1	Model 2	Model 3	Model 4
**Level 1**					
Intercept *(SD)*	13.74 *** (0.50)	15.31 *** (0.96)	16.00 *** (0.67)	16.66 *** (2.61)	15.91 *** (0.60)
Non-gamer (*SD*)		−3.01 *** (0.84)	− 3.64 *** (0.72)	−3.53 *** (0.70)	−3.52 *** (0.71)
Gamer		−	−	−	−
Procrastination (*SD*)		1.48 *** (0.53)	1.67 *** (0.31)	1.67 *** (0.31)	1.66 *** (0.31)
Learning behavior (*SD*)		−0.04 (0.06)			
Social behavior (*SD*)		−0.07 (0.04)			
Gender male (*SD*)		0.78 (0.84)			
Gender female		−			
Time spent online (*SD*)		0.25 * (0.12)	0.26 * (0.12)	0.26 * (0.12)	0.26 * (0.12)
**Level 2**					
Procrastination (school mean)				1.16 (1.34)	
Learning behavior (school mean)				−0.12 (0.19)	
Social behavior (school mean)				−0.29 * (0.14)	−0.28 ** (0.09)
Time spent online (school mean)				−0.29 (0.31)	
Education level				0.17 (0.82)	
**Model Fit Parameters**					
–2 Log-Likelihood	2843.441	2665.111	2684.213	2673.574	2677.569
AIC	2849.441	2683.111	2696.213	2695.619	2691.569
*χ^2^* (df)	7.020 **	178.33 **	−19.102 **	10.639	−3.995
Pseudo R^2^ (level 1)		14.98%	13.96%	18.36%	17.45%
Pseudo R^2^ (level 2)		12.27%	11.85%	24.66%	23.00%

Note. * *p* = 0.05; ** *p* = 0.01; *** *p* = 0.001; Maximum likelihood estimation. Level-1 parameters are groupmeancentered, level-2 parameters are grand meancentered. Pseudo-R^2^ was computed with the simplified formula by Snijders and Bosker [75]. *χ*^2^ tests for model fit were performed in comparison to the previous model. IUD: Internet use disorders; CSAS: German Video Game Dependency Scale.
